# Quantitative trait loci affecting reproductive phenology in peach

**DOI:** 10.1186/1471-2229-14-52

**Published:** 2014-02-22

**Authors:** José F Romeu, Antonio J Monforte, Gerardo Sánchez, Antonio Granell, Jesús García-Brunton, María L Badenes, Gabino Ríos

**Affiliations:** 1 Instituto Murciano de Investigación y Desarrollo Agrario y Alimentario (IMIDA), La Alberca, Murcia, Spain; 2 Instituto de Biología Molecular y Celular de Plantas (IBMCP, CSIC-UPV), Valencia, Spain; 3 Instituto Valenciano de Investigaciones Agrarias (IVIA), Moncada, Valencia, Spain

**Keywords:** *Prunus persica*, Bud dormancy, Chilling requirement, Heat requirement, Flowering, Fruit maturation, QTL

## Abstract

**Background:**

The reproductive phenology of perennial plants in temperate climates is largely conditioned by the duration of bud dormancy, and fruit developmental processes. Bud dormancy release and bud break depends on the perception of cumulative chilling and heat during the bud development. The objective of this work was to identify new quantitative trait loci (QTLs) associated to temperature requirements for bud dormancy release and flowering and to fruit harvest date, in a segregating population of peach.

**Results:**

We have identified QTLs for nine traits related to bud dormancy, flowering and fruit harvest in an intraspecific hybrid population of peach in two locations differing in chilling time accumulation. QTLs were located in a genetic linkage map of peach based on single nucleotide polymorphism (SNP) markers for eight linkage groups (LGs) of the peach genome sequence. QTLs for chilling requirements for dormancy release and blooming clustered in seven different genomic regions that partially coincided with loci identified in previous works. The most significant QTL for chilling requirements mapped to LG1, close to the *evergrowing* locus. QTLs for heat requirement related traits were distributed in nine genomic regions, four of them co-localizing with QTLs for chilling requirement trait. Two major loci in LG4 and LG6 determined fruit harvest time.

**Conclusions:**

We identified QTLs associated to nine traits related to the reproductive phenology in peach. A search of candidate genes for these QTLs rendered different genes related to flowering regulation, chromatin modification and hormone signalling. A better understanding of the genetic factors affecting crop phenology might help scientists and breeders to predict changes in genotype performance in a context of global climate change.

## Background

The timing of reproductive events of perennial plants in temperate climates is largely conditioned by dormancy, a period of cyclic quiescence during the low temperatures of autumn and winter, which protects meristems within buds from the detrimental effects of cold and water stress. The pioneering work by Lang [1] distinguished the dormancy due to mechanisms intrinsic to the bud (endodormancy) from the inhibition of growth imposed by other organs of the plant (paradormancy) or by environmental factors (ecodormancy). However, more recent reviews tend to emphasize the idea of dormancy as a state within the meristem, independently of the origin of the dormancy-imposing cues; and highlight the dynamic and quantitative nature of dormancy, varying in intensity according to intrinsic and environmental signals [2,3].

The quantitative perception of environmental chilling is the major and best-known factor favouring the release of bud dormancy [4]. The extent of chilling needed is highly genotype-dependent and constitutes an adaptive strategy to the duration of the cold season under specific climate conditions. After fulfilment of the chilling requirements for dormancy release, a period of warm temperatures is needed prior to bud burst (heat requirement) and the subsequent developmental phases leading to fruit set, growth and maturation. Both, chilling requirement for dormancy release and fruit maturation time have been described as two major limiting factors determining respectively the southern and northern boundaries to the geographical distribution of temperate species [5].

Recently researchers have increased their interest on the effects of global climate warming on plant phenology [6]. Whereas some species are expected to advance their growing season due to the increasing temperatures in spring, others could delay or experience abnormal bud burst as a result of an insufficient chilling for dormancy release in winter. In particular, a delay in the beginning of the growing season observed in the meadow and steppe vegetation of the Tibetan Plateau from the mid-1990s has been related to the later fulfilment of chilling requirements [7].

Plant species cope with changing climate conditions by shifting their geographical distribution, with a plastic response of plant phenology to environmental changes, or alternatively through the natural selection of populations with dormancy, flowering and fruit maturation traits adapted to the new conditions [8]. The knowledge on the genetic factors affecting these phenology-related traits is scarce and fragmentary in woody perennial species, although recent remarkable advances have been reached based on the comparison with analogous processes of annual model species, the use of novel transcriptomic and molecular approaches, and the exploitation of the natural variability by means of mutant and quantitative trait locus (QTL) analyses. Some of the most relevant QTL studies on reproductive phenology have been conducted in poplar and in species within the Rosaceae family. In poplar these studies have focused mostly on bud set and bud flush in spring, resulting in some loci co-localizing with genes involved in light perception and abscisic acid (ABA) signalling [9-11]. In apple, QTL analysis of bud break date in flower and vegetative buds of two different progenies pointed to two major genomic regions controlling these traits in linkage group (LG) 8 and LG9, showing numerous genes involved in cell cycle control [12]. Blooming date and fruit maturation date traits have been frequently analyzed in QTL analyses of species from the genus *Prunus*, such as almond, peach, apricot and sweet cherry [13-21]. In order to better characterize at the genetic level the physiological response of buds to chilling, some QTL studies included the chilling and heat requirement traits in their analyses [22-24]. In spite of the numerous genes proposed as putative candidates for the different QTLs described in these reports, none of them has been functionally involved in dormancy or flowering regulation, with the exception of *DORMANCY-ASSOCIATED MADS-BOX* (*DAM*) genes [25,26].

The objective of this work was to identify new QTLs and candidate genes associated to chilling and heat requirements in a *Prunus persica* [L.] Batsch (peach) population segregating from a sequential cross of three varieties with different dormancy behaviour and origin. The fruit harvest time trait was also analyzed in order to gain insight into the effect of dormancy duration on the subsequent fruit phenology.

## Results

### Phenotypic assessment and correlation between traits

**Figure 1:**
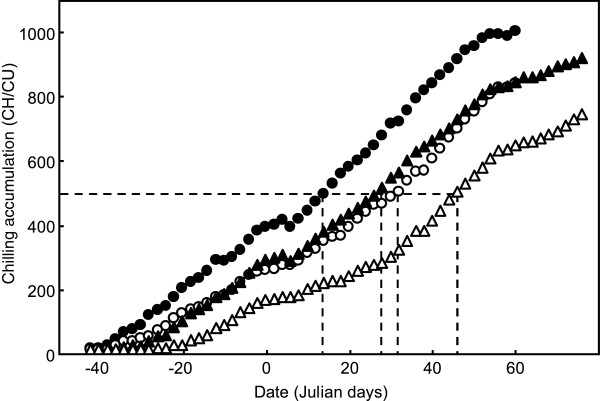
**Chilling accumulation in AA and EJ locations along the cold season.** Chilling accumulation in AA (circles) and EJ (triangles) locations, according to Weinberger (white symbols) and Utah (black symbols) models. The discontinuous line is to interpolate the date with a chilling accumulation value of 500 CH/CU.

Traits evaluated in the ‘V6’ x ‘Granada’ progeny are listed in Table 1. The mean chilling requirement values for dormancy release, measured following the most popular Weinberger (CRW), Utah (CRU) and dynamic (CRD) models, were essentially equivalent in AA and EJ, in spite of the two-weeks delay of chilling accumulation observed in EJ location with respect to AA at 500 CU and 500 CH (Figure 1). This suggests that the three models used for the evaluation of chilling requirements are essentially valid under the climatic conditions of AA and EJ sites. In close agreement with the chilling delay of two weeks in EJ, the mean endodormancy release date in this location was 15 days later than in AA (Table 1). However the mean ecodormancy release and blooming delays between EJ and AA decreased to 11 and 8 days respectively, due to the faster fulfilment of heat requirements in the warmer conditions of EJ location. Favourable temperatures also accounted for the earlier fruit harvest in EJ with respect to AA. On average, the time from dormancy release to flower and fruit development was 23 days shorter in EJ than in AA.

**Table 1:** Traits investigated in the ‘V6’ x ‘Granada’ progeny, with the mean value, standard deviation and data range over the whole population in locations AA and EJ

**Trait**	**Unit**	**Abb.**	**AA population**			**EJ population**		
			**Mean**	**SD**	**Range**	**Mean**	**SD**	**Range**
Chilling requirement for endodormancy release								
Weinberger model	Chilling hours (CH)	CRW	400	118	248–760	373	138	212–658
Utah model	Chilling units (CU)	CRU	577	154	387–960	591	145	380–856
Dynamic model	Portions	CRD	37	8	25–58	42	8	30–58
Endodormancy release date	Julian days	EnD	20	12	1–51	35	14	15–66
Ecodormancy release date	Julian days	EcD	46	13	23–64	57	12	36–73
Blooming date	Julian days	BD	67	8	58–82	75	8	57–93
Heat requirement for ecodormancy release	growing degree hours (GDH)	HREc	2500	627	908–4004	2797	835	1105–4670
Heat requirement for blooming	growing degree hours (GDH)	HRB	5110	669	2801–7372	5956	830	3826–7956
Period of time between endo- and ecodormancy release	Days	PEnEc	26	6	11–41	22	7	7–37
Period of time between endodormancy release and blooming	Days	PEnB	46	7	25–60	40	8	23–58
Harvest date	Julian days	HD	187	19	155–218	171 (2011)	13	144–192
						179 (2012)	14	150–204

Abb., variable abbreviation; SD, standard deviation.

**Figure 2:**
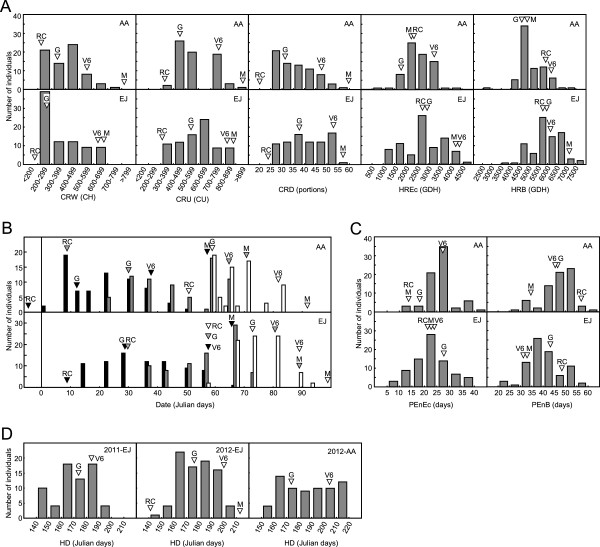
**Frequency distribution of traits measured in the ‘V6’ x ‘Granada’ progeny.** Chilling requirement (CRW, CRU and CRD) and heat requirement (HREc and HRB) variables are represented in **A**. The values for the ‘V6’ (V6), ‘Granada’ (G), ‘Red Candem’ (RC) and ‘Maruja’ (M) parents are indicated by arrows. Endodormancy release date (EnD; black bars), ecodormancy release date (EcD; grey bars), and blooming date (BD; white bars) are represented in **B**. The arrows indicating date values of parents are black for EnD, grey for EcD and white for BD. The panel **C** includes the endodormancy-ecodormancy (PEnEc) and endodormancy-blooming (PEnB) time periods. Harvest date (HD) trait is represented in panel **D**.

Variables CRW-AA, CRW-EJ, CRU-EJ, CRD-EJ, HRB-AA, EcD-AA, BD-AA, PEnB-AA and HD-2012-AA departed from normality due to altered standardized skewness or kurtosis of their frequency distribution (Figure 2). CRW-AA and CRW-EJ distributions were particularly skewed to the left, that is enriched in low chilling individuals. EcD and PEnB distributions showed bimodal or multimodal profiles, with two or more separated peaks in both locations.

The parental cultivars of ‘V6’ selection, ‘Maruja’ and ‘Red Candem’, had the highest and lowest range values of chilling requirement distributions (CRW, CRU and CRD) and date variables (EnD, EcD, BD and HD) respectively. ‘Maruja’ and ‘Red Candem’ data were not available for variables HD-2011-EJ and HD-2012-AA. However traits related to heat/time requirements for ecodormancy release and blooming (HREc, HRB, PEnEc and PEnB) showed a more variable location of parental genotypes within the range, and numerous transgressive segregants exceeded the parent values (Figure 2).

The Pearson correlation coefficients between variables are shown in Table 2. The chilling requirement (CRW, CRU and CRD), EnD, EcD and BD traits showed strong positive correlations among them (*r* ≥ 0.84, *P* < 0.01), moderate positive correlations with HD (*r* ≥ 0.30, *P* < 0.05), and strong negative correlation with PEnB. Heat requirements traits (HREc and HRB) were also correlating with their respective time interval counterparts (PEnEc and PEnB) with *r* ≥ 0.60 and *P* < 0.01. Correlations between AA and EJ locations were high for most of the traits, with the exception of HREc (*r* = 0.15, *P* = 0.24), HRB (*r* = 0.23, *P* = 0.06) and PEnEc (*r* = 0.24, *P* = 0.05) (Table 3), which indicates a strong genotype-x-environment interaction in these latter traits.

**Table 2:** Pearson correlation coefficients between variables in the ‘V6’ x ‘Granada’ progeny

**Variable**	**Location**	**CRW**	**CRU**	**CRD**	**EnD**	**EcD**	**BD**	**HREc**	**HRB**	**PEnEc**	**PEnB**	**HD-2011**
CRU	AA	0.99**										
	EJ	0.98**										
CRD	AA	0.99**	1.00**									
	EJ	0.98**	1.00**									
EnD	AA	0.99**	1.00**	1.00**								
	EJ	0.98**	1.00**	1.00**								
EcD	AA	0.87**	0.90**	0.90**	0.90**							
	EJ	0.84**	0.88**	0.88**	0.87**							
BD	AA	0.86**	0.86**	0.86**	0.86**	0.85**						
	EJ	0.87**	0.87**	0.86**	0.87**	0.88**						
HREc	AA	0.31**	0.32**	0.32**	0.31**	0.59**	0.43**					
	EJ	0.11	0.12	0.11	0.09	0.52**	0.36**					
HRB	AA	-0.01	-0.01	-0.02	-0.03	0.08	0.46**	0.33**				
	EJ	-0.09	-0.13	-0.14	-0.14	0.11	0.36**	0.58**				
PEnEc	AA	-0.03	0.03	0.03	0.02	0.45**	0.19	0.74**	0.25*			
	EJ	-0.55**	-0.50**	-0.50**	-0.52**	-0.03	-0.23*	0.72**	0.47**			
PEnB	AA	-0.80**	-0.80**	-0.80**	-0.81**	-0.64**	-0.39**	-0.05	0.60**	0.18		
	EJ	-0.83**	-0.85**	-0.85**	-0.85**	-0.61**	-0.48**	0.22*	0.63**	0.68**		
HD-2011	EJ	0.37**	0.36**	0.37**	0.36**	0.36**	0.40**	0.22	0.14	-0.07	-0.22	
HD-2012	AA	0.30*	0.30*	0.31*	0.32**	0.44**	0.32**	0.31**	-0.01	0.36**	-0.21	
	EJ	0.37**	0.38**	0.39**	0.37**	0.40**	0.37**	0.24*	0.06	-0.04	-0.26*	0.87**

*P < 0.05, **P < 0.01.

**Table 3:** Pearson correlation coefficients between variables measured in different locations (AA/EJ)

**Variable**	**AA/EJ**	***P*-value**
CRW	0.88	<0.01
CRU	0.89	<0.01
CRD	0.89	<0.01
EnD	0.89	<0.01
EcD	0.87	<0.01
BD	0.79	<0.01
HREc	0.15	0.24
HRB	0.23	0.06
PEnEc	0.24	0.05
PEnB	0.61	<0.01
HD-2012	0.96	<0.01

### Map construction

Genotyping was performed using the International Peach SNP Consortium (IPSC) peach 9 K Infinium® II array [27]. Briefly, 2,865 SNPs from the array were identified as polymorphic (40% of the total) and used for map construction. Since the different linkage groups showed several SNPs co-localizing at the same locus, one SNP per locus was selected in order to obtain a condensed map. For the ‘V6’ map, a total of 178 SNPs were retained that covered all the chromosomes but chromosome 2, representing a total distance of 480 cM with an average marker density between adjacent markers of 2.94 cM. For ‘Granada’ map, 76 SNPs were retained, covering chromosomes 2, 4, 5, 6, 7 and 8 with a total distance of 276 cM and an average marker density of 3.87 cM. This lack of polymorphic markers in large chromosome regions should be due to homozygosity of those regions. Further details on genetic map construction can be found in [28].

### QTL analysis of chilling requirement and flowering time

**Figure 3:**
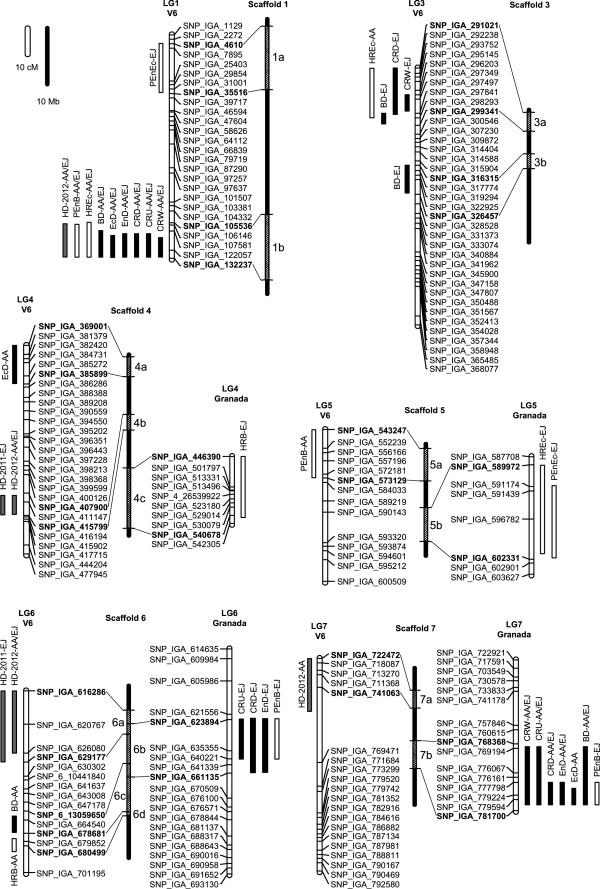
**Location of quantitative trait loci (QTLs) in the ‘V6’ x ‘Granada’ genetic map.** QTLs are labelled by boxes (two-LOD confidence interval). Boxes are black for chilling requirement and blooming variables (CRW, CRU, CRD, EnD, EcD and BD), white for heat requirement variables (HREc, HRB, PEnEc and PEnB) and grey for harvest date (HD). ‘V6’ and ‘Granada’ genetic maps are vertical white bars showing single nucleotide polymorphisms (SNP) markers. Genome scaffolds are represented as vertical black bars placed between their respective ‘V6’ and ‘Granada’ maps. The positions of SNP markers flanking the confidence intervals of QTLs are labelled in bold on genome scaffolds, limiting a genomic region named with a number (for the scaffold) and a letter (for the order of the region along the scaffold), which was used to identify putative candidate genes. Within each genomic region, QTLs of the same variable corresponding to different locations are fused in a single box. Scale bars for genetic maps and scaffolds are shown in the upper left part of the figure.

The analysis of co-segregation between SNPs and phenotypic data led to the identification of QTLs for all the investigated traits. For every variable, the LOD threshold for *P* < 0.05 calculated by the permutation test was between 2.2 and 2.5. Several major QTLs explaining 60-76% of the phenotypic variance of CRW, CRU, CRD, EnD, EcD and BD overlapped within the genomic region 1b, close to SNP_IGA_122057 marker at the end of LG1 in ‘V6’ map (Table 4, Figure 3). These QTLs in 1b were consistently detected in the AA and EJ locations.

**Table 4:** Quantitative trait loci (QTLs) detected in the ‘V6’ x ‘Granada’ progeny

**Variable**	**Location/year**	**Map**	**LG**	**Position (cM)**	**Nearest marker**	**CI (cM)**	**LOD**	**R**^**2 **^**(%)**	**Additive effect**
CRW	AA	V6	1	72.5	SNP_IGA_122057	68.2-74.5	16.3	69	-206.6
	EJ	V6	1	73.5	SNP_IGA_122057	68.2-74.5	22.3	64	-228.8
	EJ	V6	3	10.4	SNP_IGA_297497	8.9-14.4	3.8	10	-123.2
	EJ	G	7	29.9	SNP_IGA_769194	29.3-40.5	3.9	20	-159.7
	AA	G	7	45.5	SNP_IGA_779224	41.3-48.9	3.9	24	-120.1
CRU	AA	V6	1	72.5	SNP_IGA_122057	66.8-74.5	16.3	76	-284.2
	EJ	V6	1	73.5	SNP_IGA_122057	69.3-74.5	22.1	72	-248.1
	EJ	G	6	34.2	SNP_IGA_635355	23.6-37.1	3.8	18	-143.8
	EJ	G	7	29.9	SNP_IGA_769194	29.3-41.3	3.7	18	-158.5
	EJ	G	7	45.5	SNP_IGA_779224	43.2-48.9	4.5	21	-151.2
	AA	G	7	45.5	SNP_IGA_779224	41.3-48.9	3.9	25	-158.5
CRD	AA	V6	1	72.5	SNP_IGA_122057	66.8-74.5	17.3	65	-14.1
	EJ	V6	1	73.5	SNP_IGA_122057	68.4-74.5	22.1	67	-13.9
	EJ	V6	3	2.9	SNP_IGA_293752	0.0-15.6	2.5	6	-4.2
	EJ	G	6	34.2	SNP_IGA_635355	23.3-41.6	2.8	13	-7.1
	AA	G	7	45.5	SNP_IGA_779224	41.7-48.9	4.0	25	-8.4
	EJ	G	7	45.5	SNP_IGA_779224	41.3-48.9	3.2	14	-6.5
EnD	AA	V6	1	72.5	SNP_IGA_122057	66.8-74.5	17.0	64	-26.6
	EJ	V6	1	73.5	SNP_IGA_122057	67.8-74.5	20.2	63	-22.2
	EJ	G	6	34.2	SNP_IGA_635355	23.4-41.6	3.0	14	-11.9
	AA	G	7	45.5	SNP_IGA_779224	41.9-48.9	3.9	25	-12.4
	EJ	G	7	45.5	SNP_IGA_779224	41.3-48.9	3.5	16	-11.2
EcD	AA	V6	1	74.5	SNP_IGA_122057	72.5-74.5	15.9	65	-13.4
	EJ	V6	1	73.5	SNP_IGA_122057	67.4-74.5	20.2	63	-19.2
	AA	V6	4	2.8	SNP_IGA_381379	0.0-12.8	2.8	8	-8.1
	AA	G	7	45.5	SNP_IGA_779224	43.3-48.9	5.2	30	-15.3
BD	AA	V6	1	72.5	SNP_IGA_122057	65.8-74.5	16.8	60	-22.4
	EJ	V6	1	72.5	SNP_IGA_122057	67.2-73.5	19.9	60	-12.9
	EJ	V6	3	17.8	SNP_IGA_298293	15.2-18.8	5.2	16	-9.6
	EJ	V6	3	33.1	SNP_IGA_316315	32.5-41.9	3.9	11	8.1
	AA	V6	6	43.2	SNP_IGA_664540	42.2-47.7	5.1	17	-11.6
	AA	G	7	29.9	SNP_IGA_769194	29.3-41.3	2.6	19	-8.8
	EJ	G	7	29.9	SNP_IGA_769194	29.3-41.3	3.7	22	-9.8
	AA	G	7	45.5	SNP_IGA_779224	43.4-48.9	4.5	29	-9.5
	EJ	G	7	45.5	SNP_IGA_779224	41.3-48.9	3.6	20	-8.3
HREc	AA	V6	1	73.5	SNP_IGA_122057	63.1-74.5	3.0	15	-497.2
	EJ	V6	1	74.5	SNP_IGA_132237	63.7-74.5	5.4	25	-644.8
	AA	V6	3	12.4	SNP_IGA_297497	0.0-16.7	2.5	15	-253.7
	EJ	G	5	20.4	SNP_IGA_591439	2.8-32.5	3.0	17	432.3
HRB	AA	V6	6	50.6	SNP_IGA_679852	49.7-54.1	5.6	32	457.9
	EJ	G	4	11.4	SNP_IGA_513496	0.0-20.6	2.7	12	584.2
PEnEc	EJ	V6	1	14.3	SNP_IGA_7895	2.8-19.3	2.8	15	-5.9
	EJ	G	5	15.4	SNP_IGA_591439	9.8-34.1	2.7	14	-5.0
PEnB	AA	V6	1	73.5	SNP_IGA_122057	65.8-74.5	8.8	39	8.6
	EJ	V6	1	73.5	SNP_IGA_122057	63.7-74.5	8.3	33	9.0
	AA	V6	5	9.5	SNP_IGA_556166	0.0-16.2	2.9	12	4.8
	EJ	G	6	34.2	SNP_IGA_635355	23.5-37.1	4.3	18	6.5
	EJ	G	7	45.5	SNP_IGA_779224	41.3-48.9	2.6	9	4.9
HD	AA/2012	V6	1	70.8	SNP_IGA_107581	63.6-73.5	7.8	16	-16.1
	EJ/2012	V6	1	73.5	SNP_IGA_122057	65.6-74.5	7.8	15	-10.9
	AA/2012	V6	4	52.9	SNP_IGA_411147	50.5-56.9	20.1	51	-27.9
	EJ/2011	V6	4	52.9	SNP_IGA_411147	50.5-57.0	16.2	49	-19.4
	EJ/2012	V6	4	53.9	SNP_IGA_411147	52.9-56.8	21.1	54	-20.5
	AA/2012	V6	6	11.1	SNP_IGA_620767	0.0-20.9	2.8	6	10.0
	EJ/2011	V6	6	11.0	SNP_IGA_620767	0.0-23.9	3.4	8	7.7
	EJ/2012	V6	6	11.1	SNP_IGA_620767	1.0-19.9	5.4	10	8.9
	AA/2012	V6	7	4.0	SNP_IGA_713270	0.0-17.8	4.0	9	11.7

LG, linkage group; CI, two-LOD confidence interval of QTL position; LOD, logarithm of the odds; R^2^, percentage of the phenotypic variance explained by the QTL.

Another cluster of QTLs was identified in the LG7 of ‘Granada’ map. The LOD plots showed two close linked peaks, suggesting the possibility that two linked QTLs were present in this genomic region (Table 4). Given that the 2-LOD confidence intervals of these putative linked QTLs were shortly overlapping (Table 4) we considered that region as a single QTL, although the possibility of two linked QTLs could not be discarded. This region in LG7 contained QTLs for the traits chilling requirement, EnD and BD, which were consistently detected in the two locations with R^2^ ranging between 14% and 29%. Also a QTL for EcD was detected in the AA location explaining 30% of the genetic variance. Other QTLs with minor effects (R^2^ ≤ 18%) clustered in LG3 and LG6, although they were detected in only one location (Table 4, Figure 3).

### QTL analysis of heat requirement and ecodormancy release

Traits describing processes following endodormancy release were HREc and HRB, which use the Anderson model to estimate the heat requirements for ecodormancy release and blooming respectively. Two additional traits, PEnEc and PEnB, estimate the time in days required for the fulfilment of HREc and HRB values respectively, starting from the endodormancy release date. A QTL cluster for HREc and PEnB traits with consistent major effects in both locations (R^2^ = 15-39%) was detected in LG1 of ‘V6’ map, overlapping with QTLs for chilling requirement and flowering time described above. Additional QTLs with minor effects but not consistent across locations were also detected in LG3, LG4, LG5, LG6 and LG7 (Table 4, Figure 3).

### QTL analysis of fruit harvest date

QTLs for HD were detected in four genomic regions of LG1, LG4, LG6 and LG7 of the ‘V6’ map (Table 4, Figure 3). All of them were consistent across geographical locations and years, except for a minor QTL in LG7. The major QTL was located in region 4b, with LOD values between 16.2 and 21.1 and R^2^ around 50%. A second cluster of QTLs was associated to the region 1b, co-localizing with the most significant QTL for chilling requirement (CRW, CRU and CRD), EnD, EcD, BD, HREc and PEnB, which highlights the outstanding influence of this locus on the reproductive phenology of these peach cultivars. Other minor QTLs, explaining 10% or less of the phenotypic variance of HD, were localized in genomic regions 6a and 7a. The most significant QTLs in regions 1b and 4b showed negative effects on HD.

### Search of candidate genes

QTL regions 1b, 3a, 4b, 6a, 6b and 7b, containing multiple QTLs and QTLs with high significance, relevance and consistent effects among trials were searched for the presence of plausible candidate genes. The QTL regions limited by the genome coordinates shown in Additional file 1 were examined manually. Those genes showing high similarity to genes involved in molecular and physiological processes previously associated to regulation of bud dormancy, flowering and fruit maturation, such as ABA regulation, cold acclimation, ethylene signalling, chromatin modification, flowering and vernalization pathways were selected as candidate genes (Table 5). Also the transcriptional regulators ppa008979m, ppa012329m, ppa011123m (*DAM4*), ppa010822m (*DAM5*) and ppa010714m (*DAM6*) were included in Table 5 given their dormancy-dependent expression in flower buds [29-31]. These last four genes were added to the candidate list due to the outstanding importance of *DAM* genes in dormancy regulation, which were previously postulated as candidate genes in other QTL studies [22,23], and their extreme localization at the end of LG1 after the last SNP marker flanking region 1b (SNP_IGA_132237).

**Table 5:** Peach genes located in the two-LOD confidence interval of selected QTLs and other candidate genes

**QTL region**	**Peach model (gene)**	**Genomic location (Mb)**	**Blastp hit in *Arabidopsis***	**E-value**	**Description**
1b	ppa000228m	33.9	PKL	0	Chromatin remodelling factor PICKLE
	ppa008143m	34.3	COL4	2×10^-140^	Zinc finger protein CONSTANS-LIKE 4
	ppa003748m	34.8	HAB1	8×10^-177^	Protein phosphatase 2C
	ppa006590m	35.0	HDA2	0	Histone deacetylase 2
	ppa008979m	35.9	At5g67300	2×10^-95^	myb-related protein
	ppa006503m	36.2	AREB1	3×10^-121^	ABA-responsive element binding protein 1
	ppa000318m	37.0	EMF1	8×10^-44^	Embryonic flower 1
	ppa013757m	37.6	FPF1	8×10^-57^	Flowering promoting factor 1
	ppa000056m	39.8	EFS	0	Histone-lysine N-methyltransferase
	ppa001566m	40.0	SWI3C	0	SWI/SNF complex subunit SWI3C
	ppa005747m	40.8	HAM2	0	Histone acetyltransferase of the MYST family
	ppa024363m	41.0	AREB3	3×10^-45^	ABA-responsive element binding protein 3
	ppa012329m	46.0	At4g24440	3×10^-68^	Transcription initiation factor IIA subunit 2
	ppa010714m (*DAM6*)	46.3	SVP	7×10^-65^	MADS-box protein
	ppa010822m (*DAM5*)	46.4	SVP	1×10^-62^	MADS-box protein
	ppa011123m (*DAM4*)	46.4	AGL24	2×10^-60^	MADS-box protein
3a	ppa020502m	0.1	CCR2	2×10^-19^	Glycine-rich protein
	ppa004975m	0.1	SDG40	0	SET domain group 40
	ppa000162m	1.2	HAC1	0	Histone acetyltransferase of the CBP family
	ppa013609m	1.7	At1g54070	1×10^-19^	Dormancy/auxin associated family protein
	ppa004252m	2.4	HAB1	0	Protein phosphatase 2C
	ppa002515m	3.2	ABO5	0	Pentatricopeptide repeat-containing protein
4b	ppa010982m	10.4	ERF4	6×10^-45^	Ethylene responsive element binding factor 4
	ppa022739m	10.5	SPL4	3×10^-41^	Squamosa promoter binding protein-like 4
	ppa008301m	11.1	ANAC072	2×10^-122^	NAC domain-containing protein 72
	ppa002986m	12.0	DFL1	0	Indole-3-acetic acid amido synthetase
6a	ppa003113m	3.8	EIL3	0	Ethylene-insensitive3-like 3
	ppa001557m	6.8	ARF4	0	Auxin response factor 4
	ppa002082m	7.1	ARF10	0	Auxin response factor 10
6b	ppa009583m	8.7	HDT3	3×10^-44^	Histone deacetylase 2C
	ppa006053m	9.1	HDA9	0	Histone deacetylase 9
	ppa007108m	11.6	HDA8	0	Histone deacetylase 8
	ppa022266m	13.7	AREB3	2×10^-59^	ABA-responsive element binding protein 3
7b	ppa024294m	12.4	ATXR4	4×10^-140^	Histone-lysine N-methyltransferase
	ppa026273m	13.4	At5g42560	1×10^-74^	ABA-responsive protein (TB2/DP1, HVA22)
	ppa012369m	14.3	TFL1	7×10^-93^	Phosphatidylethanolamine-binding protein
	ppa015643m	14.5	CBF2	1×10^-48^	C-repeat/DRE binding factor 2
	ppa009356m	14.7	HDT3	6×10^-57^	Histone deacetylase 2C
	ppa001213m	15.4	CLF	0	Histone-lysine N-methyltransferase
	ppa002248m	15.6	ABA1	0	Zeaxanthin epoxidase
	ppa012239m	16.4	At4g37220	7×10^-25^	Cold acclimation protein WCOR413 family
	ppa001803m	16.7	DDM1	0	Chromatin remodelling factor

An alternative approach for the identification of candidate genes has been performed by reciprocal blast analysis of known genes involved in chromatin modification and flowering. Reciprocal blast analysis allowed the identification of putative orthologs of these genes in peach located within the genomic regions outlined by QTL analysis. Peach putative orthologs of genes coding for subunits of the Polycomb Repressive Complex 1 and 2 (PRC1, PRC2), Trithorax group proteins [32], histone demethylases with Jumonji (JMJ) domain, histone acetyltransferases and deacetylases [33] (Pandey et al. 2002), and flowering factors are listed in Table 6. Only those putative orthologs located within a QTL region are shown. Some candidate genes, such as ppa001213m, ppa000318m, ppa000228m, ppa000162m, ppa005747m and ppa012369m appear in both Tables 5 and 6; however some putative orthologs that are not located within one of the major QTL regions 1b, 3a, 4b, 6a, 6b and 7b, and candidate genes that have not be considered as putative orthologs by reciprocal blast analysis appear just once.

**Table 6:** **Putative orthologs in peach of *Arabidopsis *** **genes involved in chromatin modification and flowering located in QTL regions, identified by reciprocal blast analysis**

**Complex or pathway**	***Arabidopsis *** **protein**	**Gene model**	**Putative peach ortholog**	**Blastp E-value**	**Genomic location**	**QTL region**
Polycomb (PRC2)	CLF	At2g23380	ppa001213m	0	scaffold_7: 15427185 - 15434449	7b
	VIL2	At4g30200	ppa001943m	6×10^-177^	scaffold_1: 5841849 - 5849018	1a
Polycomb (PRC1)	EMF1	At5g11530	ppa000318m	2×10^-32^	scaffold_1: 37002560 - 37008224	1b
Trithorax	PKL	At2g25170	ppa000228m	0	scaffold_1: 33913537 - 33925777	1b
	ASH2R	At1g51450	ppa006686m	2×10^-131^	scaffold_4: 19402413 - 19406234	4c
JMJ domain	REF6	At3g48430	ppa000214m	0	scaffold_5: 14087553 - 14096069	5b
Histone acetylation	HAC1	At1g79000	ppa000162m	0	scaffold_3: 1240769 - 1249807	3a
	HAM2	At5g09740	ppa005747m	0	scaffold_1: 40838319 - 40843052	1b
	HDA6	At5g63110	ppa005308m	0	scaffold_5: 14461481 - 14464755	5b
Flowering	SVP	At2g22540	ppa011063m	8×10^-82^	scaffold_6: 18701564 - 18704722	6c
	LFY	At5g61850	ppa006372m	9×10^-87^	scaffold_5: 13116676 - 13119151	5b
	TFL1	At5g03840	ppa012369m	1×10^-73^	scaffold_7: 14280983 - 14282266	7b

## Discussion

In this work we have studied nine traits related to bud dormancy, flowering and fruit harvest in a hybrid population of peach. The high level of correlation between traits and the clustering of QTLs in certain map positions argue for a considerable degree of redundancy that recommends the joint analysis of traits in three major groups. Chilling requirement (CRW, CRU and CRD), dormancy release (EnD and EcD), and blooming date (BD) variables constitute a first group with high correlation values and similar QTLs. Additionally, the fact that a significant QTL for fruit harvest date also co-localizes with the major QTL for chilling requirement in LG1 indicates that chilling requirement is a primary determinant of the reproductive phenology in peach. Heat requirement HREc and HRB, and PEnEc and PEnB form a second group of traits with common features. Finally, HD trait deserves a separate discussion due to the particular contribution of fruit developmental programs to this trait, in spite of its significant correlation with chilling requirement trait.

### Candidate genes for chilling requirement, dormancy release and blooming time

QTLs for this first group of traits clustered in seven different map zones that corresponded to seven genomic regions defined by SNPs (1b, 3a, 3b, 4a, 6b, 6c and 7b). Some of these regions were identified in previous QTL works in *Prunus* species. Preceding attempts to describe loci affecting the blooming date trait in peach found QTLs co-localizing with our QTLs in regions 1b [16] and 7b [13,19], whereas more recent studies dissecting the chilling requirement and blooming time traits in apricot, peach and almond identified QTLs overlapping with our QTLs in regions 1b, 4a, 6b and 7b [22-24]. The QTL in LG7 appeared consistently in most of these reports. Our analysis pointed to the presence of two adjacent QTL clusters that could contribute to both chilling requirement and dormancy-blooming time traits in this zone (Table 4). Due to their shortly overlapping positions, these two putative clusters were fused and considered as one single QTL in the subsequent analysis (Figure 3). In almond, two QTLs were also found in adjacent positions in LG7 [24]. By assuming a high degree of synteny between almond and peach, the first QTL for flowering time identified in LG7 in almond co-localized with our QTL in region 7b, whereas the second QTL identified by these authors associated to chilling and heat requirement traits did not overlap with region 7b.

The availability of the peach genome sequence [34] facilitates the identification of candidate genes by *in silico* search of genes within QTL intervals. Following this approach, genes involved in light signalling, circadian clock, flowering regulation, cell cycle and phytohormone response were previously identified as candidate genes for bud phenology traits in poplar and apple [9-12].

Recent studies in apricot, peach and almond proposed *DAM* genes within the *evergrowing* (*evg*) locus as the most promising candidate genes for the major QTL affecting chilling requirement and blooming time in LG1 [22-24], based on the genomic location of *evg* and the abundant literature conferring *DAM* genes a relevant role in bud dormancy maintenance. *DAM1-6* genes are a set of six tandemly repeated MADS-box genes related to *SHORT VEGETATIVE PHASE* (*SVP*) of *Arabidopsis thaliana* that have been found partially deleted in the *evg* peach mutant showing non-dormant behaviour [25,35]. *DAM* genes are specifically expressed in buds and are affected differently by photoperiod and chilling signals [36]. *DAM5* and *DAM6* expression correlated with the dormancy state of buds, being higher in dormant buds and lower after the fulfilment of chilling requirements prior to dormancy release [37-39]. The expression of *DAM1*, *DAM5* and *DAM6* is also repressed during chilling stratification of the embryo, suggesting their participation in seed dormancy release mechanisms [40]. At the functional level, the heterologous expression of *DAM1* gene from leafy spurge (*Euphorbia esula*) delayed flowering in *Arabidopsis*[41], and *PmDAM6* from Japanese apricot (*Prunus mume*) led to growth cessation and bud set in poplar under environmental conditions favourable for growth [42].

Other candidates to be the major determinant of bud phenology located in genomic region 1b are listed in Table 5. Among them, we have proposed chromatin remodelling and modification factors such as PICKLE-like (ppa000228m), a putative SWI3C-like element of the SWITCH/SUCROSE NONFERMENTING (SWI/SNF) remodelling complex (ppa001566m), an HDA2-like histone deacetylase (ppa006590m), HAM2-like histone acetyltransferase (ppa005747m), an EARLY FLOWERING IN SHORT DAYS (EFS)-like histone methyltransferase (ppa000056m), and EMBRYONIC FLOWER1 (EMF1)-like component of the Polycomb Repressive Complex1 (PRC1) (ppa000318m). The vernalization mechanisms converging on the expression of *FLOWERING LOCUS C* (*FLC*) in *Arabidopsis* have been proposed to share molecular features with the chilling-dependent release of bud dormancy mediated by *DAM* genes [43,44]. The chromatin modification mechanisms involved in *FLC* regulation include synthesis of non-coding RNAs, histone acetylation, trimethylation of H3K4, methylation of H3K36 by EFS, trimethylation of H3K27 by PRC2 complex, and monoubiquitination of H2A by PRC1 among others [32,45]. Interestingly *DAM1* from leafy spurge and *DAM6* from peach are regulated at the chromatin level by demethylation of H3K4 and trimethylation of H3K27 following chilling accumulation, in a similar way to *FLC*[30,41]. In addition *DAM6* chromatin also showed chilling-dependent differences in H3 acetylation [30]. Altogether, these and other published data in chestnut [46,47] emphasize a prominent role of chromatin modifying pathways in bud dormancy mechanisms.

Other candidate genes in region 1b are putative components of the ABA signalling pathway, such as ppa003748m coding for a protein phosphatase 2C (PP2C) similar to HYPERSENSITIVE TO ABA1 (HAB1), and ppa006503m and ppa024363m coding for proteins similar to ABA-RESPONSIVE ELEMENT BINDING PROTEIN 1 (AREB1) and AREB3 [48]. Additional *HAB1*-like and *AREB3*-like genes are also found in regions 3a and 6b. HAB1 and other related PP2Cs perform a central role in the negative regulation of ABA signalling in *Arabidopsis*, which is overcome by the ABA-dependent interaction of PP2Cs with the ABA-receptor PYL5 [49]. In contrast to the well-established role of ABA in seed dormancy processes, only few molecular data support the function of ABA in promoting and maintaining dormancy in buds [2,3,50,51]. Furthermore, manipulating the expression of the poplar ortholog of *ABSCISIC ACID INSENSITIVE 3* (*ABI3*) caused alterations in bud formation and misregulation of numerous genes in buds [52].

In region 1b we have also identified putative flowering-related genes, such as ppa013757m similar to *FLOWERING PROMOTING FACTOR 1* (*FPF1*) [53], and putative regulatory genes found up-regulated in latent buds such as ppa008979m and ppa012329m [30].

Genomic regions 3a, 6b and 7b were also considered important for the chilling requirement trait. Among the candidate genes present in these regions, we found other chromatin-related factors, such as ppa001213m, the peach ortholog of *CURLY LEAF* (*CLF*), a component of the PRC2 complex involved in the trimethylation of histone H3 at lysine 27 [54]. As already proposed in previous works, PRC2 complexes could contribute to bud dormancy release in *Prunus* species [55,56], and more specifically to H3K27 trimethylation observed in *DAM6* concomitantly with gene down-regulation [30]. The genes ppa004975m and ppa024294m codify for other putative histone methyltransferases containing the SET-domain, with similarity to *Arabidopsis* SDG40 and ATXR4 respectively. In region 7b, ppa001803m codes for a putative SWI2/SNF2 chromatin-remodelling ATPase similar to DDM1, which makes the heterochromatin bound to histone H1 accessible to DNA methyltransferases [57].

Further histone acetyltransferases and deacetylases are localized in regions 3a, 6b and 7b. Among them, ppa009583m and ppa009356m show similarity to *HDT3* gene, coding for a histone deacetylase that modulates the ABA response [58]. Other putative elements of ABA signalling, ABA biosynthesis and stress response pathways in peach are ppa004252m, ppa002515m, ppa022266m, ppa026273m, ppa015643m, ppa002248m and ppa012239m.

### Candidate genes for heat requirement

In contrast to the well-established genetic component of chilling requirements and the close relationship between flowering time and chilling requirements described so far, the genetic control of heat requirements in *Prunus* species has been a matter of discussion in the bud dormancy field. Couvillon & Erez [59] considered the variations in heat requirement to be due to excessive chilling and found no genetic differences in heat requirements among cultivars. However, the negative correlation found previously between chilling and heat requirements has been argued to suggest the existence of a potential contribution of genetic factors to the heat requirement trait [23,60]. We have observed a similar negative correlation of PEnEc and PEnB with chilling requirement variables in this work; however HREc and HRB traits were not related significantly to chilling requirements with the exception of a positive correlation found between HREc and CRW/CRU/CRD in AA location (Table 2).

We found 13 significant QTLs for HREc, HRB, PEnEc and PEnB, located in nine different genomic regions (Table 4, Figure 3). Seven QTLs overlapped with chilling requirement QTLs in regions 1b, 3a, 6b and 7b. No coincidences with previous reports were observed, with the exception of QTLs in the genomic region 1b [23].

In addition to candidate genes proposed for genomic regions containing chilling requirement QTLs, commented in the previous section, a reciprocal blast analysis for the search of peach genes orthologous to chromatin and flowering genes from *Arabidopsis* resulted in the candidate gene list presented in Table 6. The transcript model ppa001943m, located in region 1a, was a putative ortholog of *VERNALIZATION INSENSITIVE 3-LIKE 2* (*VIL2*), coding for a component of PRC2 complexes involved in flowering under non-inductive conditions through the epigenetic regulation of the floral repressor *MADS AFFECTING FLOWERING 5* (*MAF5*) [61]. In region 4c we identified a putative ortholog of *ARABIDOPSIS ASH2 RELATIVE* (*ASH2R*) gene, a regulator of flowering time required for H3K4 trimethylation and the proper expression of *FLC* and *FLC* homologs [62]. Finally, in region 5b we identified the putative orthologs of the chromatin regulators *RELATIVE OF EARLY FLOWERING 6* (*REF6*) and *HDA6*[63,64], and the floral modulator *LEAFY* (*LFY*) [65].

### Candidate genes for fruit harvest date

The analysis of the harvest date trait resulted in a major QTL in region 4b showing numerous precedents in related works in peach and apricot [16,19,20]. The transcript model ppa010982m, similar to *ETHYLENE RESPONSIVE ELEMENT BINDING FACTOR 4* (*ERF4*) from *Arabidopsis*, has been already proposed as a candidate gene for fruit maturation date in different *Prunus* species [20]. ERF4 is a transcriptional repressor modulating ethylene and ABA responses in *Arabidopsis*[66]. In peach different *ERF* genes have been found up-regulated in ripening fruit [67], whereas similar ERFs have been postulated to be involved in fruit ripening regulation in apple [68].

On the same region 4b, the gene ppa022739m codes for a putative transcription factor containing the Squamosa-Promoter Binding Protein (SBP) domain, present in the tomato fruit ripening factor COLORLESS NON-RIPENING (CNR) [69].

However, recent fine mapping of a locus controlling maturity date in two segregating populations of peach limited the search to a 220 kb stretch within region 4b. The maturity date locus co-segregated with an indel into the gene ppa008301m coding for a NAC type transcription factor, which points to this gene as a firm candidate for controlling ripening time in peach [70].

In region 6a we should emphasize the presence of ppa003113m gene, with similarity to *ETHYLENE-INSENSITIVE3-LIKE 3* (*EIL3*), involved in regulation of the sulfur-limitation response in *Arabidopsis*[71] and similar to elements of the ethylene pathway. Other candidate genes in regions 4b and 6a were hypothetically related to auxin synthesis and response (ppa002986m, ppa001557m and ppa002082m), since auxin is known to be involved in fruit set and ripening [72].

## Conclusions

This work was aimed at the identification of genetic factors conditioning the phenological behaviour of peach. We have identified QTLs for nine traits related to bud dormancy, flowering and fruit harvest in a hybrid population of peach in two different locations. QTLs were located in a SNP-based genetic linkage map. A search of candidate genes for these QTLs rendered different genes related to flowering regulation, chromatin modification and hormone signalling. Additional studies including the characterization of proposed candidate genes in germplasm collections and functional approaches are required to identify the genes involved in dormancy, blooming and fruit maturation among these lists of candidate genes. The characterization of natural alleles of these genes might offer molecular tools to predict the potential performance of different *Prunus* species and cultivars under changing climatic conditions.

## Methods

### Plant material

The plant material used in the study was a progeny of 107 individuals derived from a cross carried out during 2008 between the F1 selection ‘V6’ (named MxR_01 in [73]) and the Brazilian non-melting peach cultivar ‘Granada’. ‘V6’ selection was derived from a cross performed in 2005 between the Spanish non-melting peach cultivar ‘Maruja’ (high chilling requirement and late ripening) and the North-American melting peach cultivar ‘Red Candem’ (low chilling and early ripening). Two individuals per genotype were grafted on ‘Garnem’ (hybrid almond x peach) rootstock, and then cultivated in the experimental orchards Agua Amarga (AA; 38° 18’ 41” N 1° 31’ 31” W, 344 m over sea level) and El Jimenado (EJ; 37° 45’ 31” N 1° 01’ 35” W, 80 m over sea level), both of them situated in the Region of Murcia, at the southeast of Spain. The experimental orchards represented different climatic conditions regarding chilling and heat accumulation, maximum, minimum and medium temperatures and humidity during winter and spring. From the 107 progeny trees 86 genotypes were grown at EJ, 74 at AA, and 70 genotypes were common to both locations. Horticultural practices such as pruning, irrigation, fertilization and control of weeds, insects and diseases were consistently performed at both orchards.

### Phenotypic assessment

Chilling requirements, heat requirements and blooming dates were evaluated in the winter and spring of 2012; fruit harvest dates were measured in the spring of 2011 and 2012. To assess chilling requirements for endodormancy release, nine one-year old shoots per genotype with a length of 20-25 cm were picked weekly. Groups of three shoots were placed in bottles containing distilled water with 3% sucrose, and incubated in a growth chamber subject to 12 h photoperiod at 22°C. The basal end of shoots was cut and the water renewed once per week. We considered that endodormancy was completed when the percentage of buds that reached the green stage (stage C) according to the Baggiolini code [74] was higher than 30% in the three groups of shoots after 10 days. It was difficult to obtain higher percentages of bud break under the artificial conditions (cutting shoots) of the laboratory. Quantification of chilling accumulation at the dormancy release date was performed using the three most common models: Weinberger [75], Utah [76] and dynamic model [77,78]. Hourly air temperatures were recorded from beginning of winter to harvest date by the SIAM station [79] and by temperature sensors (Testo T174).

To evaluate ecodormancy release and blooming time, the main phenological stages [74] were visually identified on the field weekly, from the beginning of winter to fruit set in 2011-2012. The different phenological stages were assessed quantitatively based on the ratio of buds. Thus, we considered that ecodormancy was released when 50% of buds had reached the green stage (stage C) in the field, and a percentage of 50% of open flowers (stage F) served to establish blooming date. Both dates were further expressed as Julian days and periods between endodormancy, ecodormancy and blooming time were calculated.

Heat requirements were calculated as the growing degree hours (GDH) accumulated from the release of endodormancy to the ecodormancy release and blooming dates following the Anderson model [80]. The harvest date was determined *in situ* based on fruit colour and firmness.

### Statistical analyses

Statistical analyses were performed using the Statgraphics 5.1 package (Statpoint Technologies, Warrenton, VA, USA). All correlations between traits were calculated using the Pearson coefficient. Correlations between traits in the EJ and AA locations employed exclusively those genotypes present in both locations. Departure from the normal distribution of traits was assessed by the calculation of skewness and kurtosis of frequency distributions.

### SNP genotyping and map construction

DNA from the parentals and progeny were extracted from 50 mg of leaf tissue following the method of Doyle & Doyle [81]. The concentration of DNA was checked by comparison with standard DNA ladders in agarose gels and with Quant-iTTM PicoGreen H Assay (Life Technologies, Grand Island, NY, USA). Samples were genotyped using the International Peach SNP Consortium (IPSC) peach 9 K Infinium® II array [27] at the Genotyping and Genetic Diagnosis Unit (Health Research Institute, INCLIVA, Valencia, Spain). The SNP array includes information about the physical position of all the SNPs in 9 genome scaffolds, being the first eight ones corresponding to the eight peach chromosomes. After visual inspection of genotype calls, monomorphic SNPs and SNPs with more than 5% of missing data were removed. The map construction has been described in [28] and it will be published with further details elsewhere. Briefly, we followed the two-way pseudo-test cross approach [82]. Homozygous SNPs in one parent and heterozygous in the other parent were selected to generate a genetic map for each parent, discarding SNPs heterozygous for both parents, as these markers were not used for QTL mapping and an integrated map was not necessary because the physical position of the SNPs was already known. A total of 1,970 SNPs segregated (1:1) for the ‘V6’ parent and 895 for ‘Granada’. From this data set, we removed the SNPs that showed exactly the same genotypic segregation to obtain a non-redundant and simplified map more suitable for QTL mapping. Marker data was coded as cross-pollinator (CP) and linkage analysis was performed with JoinMap® 4 [83] with a minimum LOD from 6.0 to 8.0. Map construction was performed using the regression mapping algorithm [83] and the default JoinMap® parameters (Rec = 0.40, LOD = 1, Jump = 5.0, and ripple = 1). The genotyping data was re-coded as pseudo back-cross and the order of the markers was double checked with Mapmaker 3.0 [84]. The Kosambi mapping function was used to convert recombination frequencies into map distances. The maps for each parent were drawn with MapChart 2.2 [85].

### QTL analysis

In order to facilitate computer analysis, the genetic linkage was condensed, eliminating SNPs that mapped in the same position or very close (i.e. less than 2 cM). The maps from each parent were analyzed independently and coded as two independent backcross populations. QTL analysis was performed with WinQTLcartographer 2.5 [86] by Composite Interval Mapping (CIM), and the LOD threshold to declare a QTL significant at P < 0.05 was calculated by a permutation test for the whole genome [87], implemented in WinQTLcartographer. A two-LOD support interval was taken as a confidence interval for the detected QTLs. That is, the confidence intervals were limited by a decrease of two in the LOD score at both sides of the QTL peak.

### Candidate gene selection

By using the BioMart tool in the phytozome web-page [88] we obtained the annotated transcript models contained between the SNP markers flanking the QTL regions (Additional file 1 and Additional file 2). These markers included the two-LOD confidence interval of their respective QTL. Those genes similar to known genes involved in ABA regulation, cold acclimation, ethylene signalling, chromatin modification, flowering and vernalization pathways within the most relevant regions 1b, 3a, 4b, 6a, 6b and 7b were selected as candidate genes.

In order to identify putative orthologs in peach of *Arabidopsis* genes related to chromatin modification and flowering pathways we performed a reciprocal blast analysis at phytozome [88]. First we made a blastp similarity search by using the protein sequence of selected genes as query. The first hit in the peach genome was subsequently compared with the *Arabidopsis* genome by blastp search, and those genes found reciprocally by the searches in both the peach and *Arabidopsis* genomes were considered as putative orthologs.

## Competing interests

The authors declare that they have no competing interests.

## Authors’ contributions

JFR carried out the genetic studies and helped to draft the manuscript. AJM carried out the linkage map construction and QTL analysis. GS participated in the linkage map construction and QTL analysis. AG participated in the design and coordination of the study. JGB participated in the design and coordination of the study. MLB conceived the study, and participated in its design and coordination. GR carried out the candidate gene search and drafted the manuscript. All authors read and approved the final manuscript.

## Supplementary Material

Additional file 1 Name and genomic position of SNPs flanking the QTL regions. Click here for file

Additional file 2 Annotation of genes within the QTL regions. Click here for file
